# Interplay Between the Autophagy-Lysosomal Pathway and the Ubiquitin-Proteasome System: A Target for Therapeutic Development in Alzheimer’s Disease

**DOI:** 10.3389/fncel.2018.00126

**Published:** 2018-05-09

**Authors:** Hianara A. Bustamante, Alexis E. González, Cristobal Cerda-Troncoso, Ronan Shaughnessy, Carola Otth, Andrea Soza, Patricia V. Burgos

**Affiliations:** ^1^Institute of Physiology, Faculty of Medicine, Universidad Austral de Chile, Valdivia, Chile; ^2^Center for Interdisciplinary Studies on the Nervous System (CISNe), Universidad Austral de Chile, Valdivia, Chile; ^3^Fundación Ciencia y Vida, Santiago, Chile; ^4^Centro de Biología Celular y Biomedicina (CEBICEM), Facultad de Medicina y Ciencia, Universidad San Sebastián, Santiago, Chile; ^5^Center for Aging and Regeneration (CARE), Facultad de Ciencias Biológicas, Pontificia Universidad Católica de Chile, Santiago, Chile; ^6^Institute of Clinical Microbiology, Faculty of Medicine, Universidad Austral de Chile, Valdivia, Chile

**Keywords:** Alzheimer’s disease, ubiquitin, proteasome, autophagy, lysosomes, HSV-1, neuronal dysfunction, proteostasis

## Abstract

Alzheimer’s disease (AD) is the most common cause of age-related dementia leading to severe irreversible cognitive decline and massive neurodegeneration. While therapeutic approaches for managing symptoms are available, AD currently has no cure. AD associates with a progressive decline of the two major catabolic pathways of eukaryotic cells—the autophagy-lysosomal pathway (ALP) and the ubiquitin-proteasome system (UPS)—that contributes to the accumulation of harmful molecules implicated in synaptic plasticity and long-term memory impairment. One protein recently highlighted as the earliest initiator of these disturbances is the amyloid precursor protein (APP) intracellular C-terminal membrane fragment β (CTFβ), a key toxic agent with deleterious effects on neuronal function that has become an important pathogenic factor for AD and a potential biomarker for AD patients. This review focuses on the involvement of regulatory molecules and specific post-translational modifications (PTMs) that operate in the UPS and ALP to control a single proteostasis network to achieve protein balance. We discuss how these aspects can contribute to the development of novel strategies to strengthen the balance of key pathogenic proteins associated with AD.

## Introduction

Alzheimer’s disease (AD) is the most common age-related neurodegenerative disorder associated with a progressive decline in brain function due to the accumulation of harmful deleterious proteins resulting in neuronal degeneration and death. One fundamental, largely unsolved question in this field is how different cellular mechanisms coordinate to avoid damage by toxic protein accumulation. In this regard, during the aging process it is known that the activity of the ubiquitin proteasome system (UPS) and the autophagy-lysosomal pathway (ALP) declines, two of the major catabolic pathways of eukaryotic cells. As a consequence, cells become overloaded with deleterious proteins, contributing to the development of age-related diseases such as AD (Tai and Schuman, 2008; López-Otín et al., 2013; Morimoto and Cuervo, 2014; Saez and Vilchez, 2014; Vilchez et al., 2014). The UPS is responsible for the degradation of most cytosolic and nuclear proteins, including short- and long-lived proteins (Collins and Goldberg, 2017) as well as aberrant proteins, whereas the ALP is specialized in degrading protein aggregates together with damaged organelles. A common feature of the two processes is the attachment of polyubiquitin conjugates to specific cargo proteins that initiate these degradative processes (Kwon and Ciechanover, 2017). Protein ubiquitylation is achieved by the concert action of three different enzymes, namely E1 ubiquitin-activating enzymes, E2 ubiquitin-conjugating enzymes and E3 ubiquitin ligase enzymes (Kwon and Ciechanover, 2017). Ubiquitylation is a reversible reaction as there are also several enzymes called deubiquitinating enzymes (DUBs) that can remove ubiquitin (Ub) moieties and are important regulators of the ubiquitin system (Mevissen and Komander, 2017). Specific Ub-linkages referred to as the “ubiquitin code” mediates substrate degradation by either the UPS or ALP, a code that is recognized by specific receptor proteins. These receptors bind ubiquitylated substrates via specific ubiquitin-binding domains (UBDs) and decide the fate of substrates. Interestingly, recent evidence proposes that monomeric or oligomeric physical states of these receptors, rather than specific ubiquitin moieties on the substrate, play key regulatory roles in substrate recognition (Lu et al., 2017a,b). An interesting field for exploration is how post-translational modifications (PTMs) regulate the physical state of these receptors. Recent findings have begun to explore the contribution of phosphorylation and ubiquitylation in this process, opening a completely new view of novel regulatory steps in the UPS and ALP. This review focuses on the cellular mechanisms of the UPS and ALP, common molecular aspects between these two pathways and their role in brain function. We discuss the function of attractive targets that should be considered for the development of novel therapeutic strategies.

## The Ubiquitin Proteasome System (UPS)

### Ubiquitin System

Ubiquitin is a 76-amino acid protein of 8.5 kDa that can covalently bind to lysine (K) or N-terminal residues of target proteins, a process known as ubiquitylation (Komander and Rape, 2012). After conjugation of the first ubiquitin to the substrate, other ubiquitin molecules are incorporated by attachment on ubiquitin itself through its seven K residues (K6, K11, K27, K29, K33, K48, or K63), forming different types of ubiquitin chains (Swatek and Komander, 2016). The most abundant ubiquitin chains in cells are linked to K48 and K63 (Komander and Rape, 2012; Swatek and Komander, 2016). Initially it was proposed that K48 and K63-linked polyubiquitin chains direct proteins to the proteasome and to the ALP for degradation, respectively (Korolchuk et al., 2010). However, recent findings have shown that autophagy receptors can also recognize K48-linked chains (Lu et al., 2014; Wurzer et al., 2015). Moreover, it was reported that purified proteasomes bind K63 and K48 Ub chains with similar affinities (Peth et al., 2010), in both cases triggering their rapid degradation (Hofmann and Falquet, 2001). However, proteasome inhibition accumulates different types of Ub-linkages, except for K63 ubiquitin-tagged proteins, suggesting either that ubiquitin K63-tagged proteins are not substrates of the proteasome (Xu et al., 2009) or that they are not accumulated because of activation of the ALP in response to proteasome inhibition.

Proteins are tagged with ubiquitin conjugates through a sequential enzymatic mechanism involving three distinct classes of enzymes (Ciechanover and Kwon, 2015). In the first step of the ubiquitin cascade, the ubiquitin-activating enzyme (E1) activates the glycine residue at the C-terminal of the ubiquitin molecule in an ATP-dependent manner. Three types of E1 enzymes have been described. Subsequently, activated ubiquitin is then transferred to a cysteine residue of the ubiquitin-conjugating enzyme (E2), encoded by at least 37 genes. Finally, the ubiquitin ligase enzyme (E3) links the ubiquitin from the E2 enzyme by its C-terminal to the ε-amino group of a K residue on the target protein. To date, the number of E3 ubiquitin ligases has reached almost one thousand, conferring specificity among a versatile range of substrates (Buetow and Huang, 2016). Interestingly, and similar to other PTMs, ubiquitylation is reversible due to the action of DUBs that hydrolyze the isopeptide bond between the ubiquitin C-terminus and the substrate, as well as ubiquitin-ubiquitin covalent bonds. Approximately 100 different kinds of DUBs have been identified in the human genome presenting distinct mechanisms of action, including cleavage of polyubiquitin chains, recycling of ubiquitin prior to substrate degradation and reversal of ubiquitin conjugation, among others (Mevissen and Komander, 2017). The proteasome itself has three DUBs that allow for efficient substrate degradation, one of them intrinsic to the proteasome and the other two associate transiently to the proteasome (Borodovsky et al., 2001; Verma et al., 2002; Yao and Cohen, 2002; Stone et al., 2004). Similarly, DUBs have garnered attention in the field of autophagy as key regulators of efficient protein degradation (Drießen et al., 2015; Xu D. et al., 2016).

### The 26S Proteasome System

The 26S proteasome is formed by two different complexes; the 20S core particle (CP) and the 19S particle (RP; Coux et al., 1996). Moreover, the 20S CP is a cylinder-like structure that comprises 28 protein subunits that are arranged in four heptameric rings. The two external rings are composed of seven different α subunits (α 1–7), whereas the two internal rings are composed of 7 different β subunits (β 1–7). The α-subunits bind to the 19S RP, regulating the selectivity and entry of specific substrates into the proteolytic chamber. The proteolytic chamber is composed of the β subunits. Three of the seven β subunits—β1, β2 and β5—have proteolytic activities, namely caspase-like, trypsin-like and chymotrypsin-like activity, hydrolyzing proteins with acidic, basic and hydrophobic peptide bonds, respectively (Kish-Trier and Hill, 2013; Dikic, 2017).

The 19S RP binds to one or both ends of the 20S CP, forming the 26S or 30S proteasome complexes, respectively (Finley, 2009), which promotes ATP- and ubiquitin-dependent protein degradation (Deveraux et al., 1994). The 19S RP is a large complex composed of two heteromeric subcomplexes termed the “base” and the “lid” (Glickman et al., 1998; Lander et al., 2012). The base is formed by six Adenosine triphosphate hydrolases (ATPases; Rpt1–Rpt6) and four non-ATPase subunits (Rpn1, Rpn2, Rpn10 and Rpn13).

Furthermore, the base recognizes ubiquitin-tagged proteins through Rpn1, Rpn10 and Rpn13—key proteasome ubiquitin receptors—a step that is sequentially followed by the unfolding and translocation of the substrates through the proteolytic chamber of the 20S proteasome (Deveraux et al., 1994; Husnjak et al., 2008; Rabl et al., 2008; Tomko et al., 2010; Shi et al., 2016; Yu and Matouschek, 2017). On the other hand, the lid contains nine non-ATPase subunits, where its main function is the deubiquitylation of the entering substrates (Verma et al., 2002). For this task, the lid contains the metalloprotease Rpn11, an enzyme with DUB activity (Verma et al., 2002; Yao and Cohen, 2002) that catalyzes the deubiquitylation of the vast majority of proteins that enter the proteolytic chamber of the 20S proteasome, a crucial step in proteasomal degradation. In addition to Rpn11, two other DUBs, the cysteine proteases Usp14 and Uch37/UchL5, contribute to the step of deubiquitylation by associating to the proteasome in a transient manner via the 19S RP (Borodovsky et al., 2001; Stone et al., 2004; Yu and Matouschek, 2017).

On the other hand and in addition to Rpn1, Rpn10 and Rpn13, other types of receptors have been characterized as key regulators of proteasome function called hHR23 (A and B), Ddi1, Ubiquilin-1 and Ubiquilin-2 (Kim et al., 2008; Rothenberg and Monteiro, 2010). These receptors share an N-terminal Ub-like domain (UBL) and a C-terminal Ub-associated domain (UBA) that allow them to bind simultaneously to the 26S proteasome and Ub conjugates, thus contributing to the recognition of substrates that are further delivered to the proteolytic chamber of the 20S proteasome (Collins and Goldberg, 2017).

### Transcriptional and Post-translational Control of the 26S Proteasome

Multiple mechanisms regulate the 26S proteasome, including transcriptional control and PTMs of proteasomal subunits (Murata et al., 2009; Schmidt and Finley, 2014). At the transcriptional level, two transcriptional factors—Nuclear respiratory factor 1 (Nrf1) and Nuclear factor erythroid 2 (Nrf2)—have been described. Nrf1 regulates the expression of genes that encode subunits of both the 20S CP and the 19S RP in cells treated with proteasomal inhibitors promoting the proteasome recovery pathway (Radhakrishnan et al., 2010). Interestingly, Nrf1 expression is upregulated upstream by mammalian rapamycin complex 1 (mTORC1) activity to ensure the availability of an intracellular pool of amino acids and thereby controlling newly synthesisized proteins (Zhang Y. et al., 2014). On the other hand, Nrf2, which has high homology, but is functionally unrelated to Nrf1, has been implicated in mediating proteasome activity (Jang et al., 2014). Indeed, when faced with oxidative stress Nrf2 upregulates the expression of proteasomal genes for the turnover of oxidized proteins (Pickering et al., 2012; Jung et al., 2014).

At least 14 different types of PTMs have been identified on proteasome subunits (Cui et al., 2014), including phosphorylation, oxidation, acetylation, glycosylation, ubiquitylation and myristoylation, among others (Schmidt and Finley, 2014; Tsakiri and Trougakos, 2015). The most common PTM is phosphorylation, which affects proteasome assembly, localization and proteolytic activity (Cui et al., 2014). More than 300 proteasome phosphorylation sites have been described, but the biological functions of most of them remain unknown (Guo et al., 2016). So far only few kinases are known to phosphorylate the 26S proteasome. In particular, phosphorylation of the α7 subunit on the residues serine (S)243 and S250 of the 20S proteasome by Casein Kinase II (CK2), a serine/threonine kinase that regulates the assembly and opening of the 26S proteasome and thus increases its activity (Castaño et al., 1996; Mason et al., 1996). Ca2^+^/calmodulin-dependent protein kinase II (CaMKIIα), which is activated by Ca^2+^ influx in response to synaptic stimuli phosphorylates the Rpt6 subunit on S120 of the 19S RP and enhances proteasomal activity without altering its expression (Djakovic et al., 2009; Jarome et al., 2016). Moreover, CaMKIIα activation promotes re-localization of the proteasome to dendritic spines in neurons (Bingol et al., 2010). On the other hand, the dual-specificity-tyrosine-regulated Kinase 2 (DYRK2), which phosphorylates the Rpt3 subunit on threonine (T)25 of the 19S RP, promotes proteasome activation in breast cancer cells and is necessary for cell proliferation (Guo et al., 2016). Furthermore, phosphorylation of the Rpt6 subunit of the 19S RP by protein kinase A (PKA; Zhang et al., 2007) or CaMKIIα (Djakovic et al., 2009) is needed for correct interaction with the α2 subunit of the 20S CP, and thus for successful assembly of the 26S proteasome (Satoh et al., 2001). Moreover, PKA-mediated phosphorylation increases 26S proteasomal assembly and activity (Asai et al., 2009; Guo et al., 2017), whereas de-phosphorylation *in vitro* reduces total proteasomal activity (Mason et al., 1996). In fact, activation of the PKA through increased cAMP levels enhances phosphorylation of Rpn6 and Rpt6 subunits on S14 and S120 of the 19S RP, respectively, and both phosphorylations enhance proteasome activity (Zhang et al., 2007; Lokireddy et al., 2015). To date few kinases have been implicated as negative regulators of proteasomal function. Among them are the non-receptor tyrosine kinases c-Abl and Arg, which induce phosphorylation of the α4 subunit on tyrosine (Y)106 and Y153 of the 20S proteasome (Liu et al., 2006; Li et al., 2015), as well as the constitutive activation of p38 Mitogen-activated protein kinase 1 (MAPK1) by activated MAPK kinase 6 (MKK6EE), which induces phosphorylation of the Rpn2 subunit on T273 (Lee S. H. et al., 2010). In summary, proteasome activity and assembly is finely regulated by phosphorylation offering an attractive niche for therapeutic intervention.

### General Aspects of Proteasome Function in the Brain

The role of the UPS has been extensively studied in the brain. The UPS regulates several aspects of brain function including synaptic plasticity, growth and development of immature neurons (Yi and Ehlers, 2007), and the abundance of proteins in the postsynaptic density involved in the postsynaptic response (Ehlers, 2003; Bingol and Schuman, 2004; Tai and Schuman, 2008). Moreover, the UPS regulates presynaptic vesicle release (Willeumier et al., 2006; Yao et al., 2007), neuronal development and synapse plasticity (Patrick, 2006). Additionally, inhibition of proteasome activity causes impairment of synaptic plasticity in animals (Lopez-Salon et al., 2001; Fonseca et al., 2006; Jarome et al., 2011). Intriguingly, in response to neuronal activity the proteasome localization switches from dendritic shafts to synaptic spines (Bingol and Schuman, 2006). Consistent with these findings proteasome activity is enhanced in the amygdala after fear learning by CaMKIIα phosphorylation of the Rpt6 subunit leading to the activation of the N-methyl-D-aspartate receptor (NMDAR; Jarome et al., 2011). On the other hand, proteasomal function confers neuronal protection against misfolded and damaged proteins, eliminating them with high efficiency (Goldberg, 2003).

### Evidence of Proteasome Dysfunction in Aging and AD

One hallmark of aging is a decrease in proteasome activity (López-Otín et al., 2013; Morimoto and Cuervo, 2014; Saez and Vilchez, 2014). In particular, the frequent detection of ubiquitin-positive aggregates in post-mortem brains of AD patients suggests that proteins in these aggregates are marked for degradation but not efficiently removed (Mayer et al., 1996; Ross and Poirier, 2004). Since the UPS becomes defective, it is logical to assume that this contributes to the accumulation of ubiquitylated and toxic proteins. The question that is commonly asked is: Which comes first, the malfunction of the UPS or the accumulation of toxic proteins that inhibit the UPS? Some studies suggest that it can be both (Ciechanover and Brundin, 2003). For example, in AD brains proteasome activity is decreased due to a reduction in trypsin and chymotrypsin-like activities (Lopez-Salon et al., 2003; Mishto et al., 2006). Consistent with these findings, pharmacological inhibition of the proteasome leads to increased levels of β-amyloid peptides (Aβ) and tau (Tseng et al., 2008), which contribute with the extracellular deposits known as amyloid plaques consisting of Aβ (Masters et al., 1985) and the formation of intracellular neurofibrillary tangles (NFTs) composed of hyperphosphorylated tau (Braak et al., 1986), two AD pathological hallmarks. Interestingly, Aβ and hyperphosphorylated tau have been shown to block the UPS by physical interaction (Mayer et al., 1996; Almeida et al., 2006; Tseng et al., 2008; Park et al., 2009; Lee M. J. et al., 2013). Moreover, the proteasome has also been shown to contribute to the degradation of the amyloidogenic C-terminal fragment CTFβ (Bustamante et al., 2013), an emerging pathogenic factor in AD (described below).

Two key proteins of UPS function, Rpn10 and Ubiquilin-1, are diminished during aging (Stieren et al., 2011; Scott et al., 2016), which could explain in part the reduction in proteasomal function and could be a possible link with AD. In this regard, mole rats, which are known to have a maximum lifespan of 28.3 years, compared with mice that live up to 3.5 years, have increased proteosomal function due to higher expression of chymotrypsin-like (β5) proteolytic activity (Pérez et al., 2009). In concordance with these results, overexpression of the β5 catalytic subunit in human fibroblast cell lines increases proteasome activity and expands the lifespan of primary IMR90 fibroblasts by 15%–20% (Chondrogianni et al., 2005). In contrast, decreased proteasomal β5 activity in transgenic mice correlates with a shortened life span and the development of age-related phenotypes (Tomaru et al., 2012). Therefore, further studies are needed to find novel therapeutic target opportunities focusing on positive regulators of the UPS.

### Autophagy-Lysosomal Pathway Molecular Players

The ALP involves the biogenesis of a unique organelle enclosed by a double lipid bilayer, named the autophagosome. The basal formation of autophagosomes occurs constitutively to regulate cellular homeostasis and different physiological processes (Reggiori and Klionsky, 2002; Mizushima and Levine, 2010; Deretic et al., 2013; Yonekawa and Thorburn, 2013). Nevertheless, the ALP is highly inducible by environmental changes and stress stimuli, such as nutrient starvation, growth factors, pathogen infection, protein aggregates or damaged organelles, working as a highly dynamic process that can resolve a variety of cellular demands (Murrow and Debnath, 2013). Autophagosomes engulf cytoplasmic constituents such as protein aggregates and damaged organelles (Khaminets et al., 2016), and later fuse with lysosomes to form a hybrid organelle called the autolysosome that mediates the degradation of the initially-selected cargo (Mizushima et al., 2008). Autophagosomes can also fuse with endosomes forming an intermediate organelle called the amphisome that, in a similar way to autophagosomes, fuses with lysosomes (Yi and Tang, 1999; Fader and Colombo, 2009; Patel et al., 2013). Whether amphisomes represent a mechanism to eliminate damaged endosomes or to eliminate the cargo that is selected in these organelles is still unclear. Moreover, whether endosomes contribute or not to the initial stages of autophagosome formation remains to be elucidated. Interestingly, depletion of endosomal sorting complexes required for transport (ESCRT) components—essential machinery for multivesicular body (MVB) biogenesis—accumulates autophagosomes. Moreover, ESCRTs are required for efficient fusion of autophagosomes with endosomes and lysosomes (Filimonenko et al., 2007; Oshima et al., 2016; González et al., 2017). In agreement with these observations, impaired ESCRT function leads to the accumulation of protein aggregates in the cytoplasm, which are decorated with ubiquitin and the autophagy receptor p62/SQTM1 (Filimonenko et al., 2007). Altogether, this strongly suggests that MVBs contribute to the efficient clearance of these aggregates through the ALP.

### ALP Regulation Through Signaling Events and GTPases

A multitude of different signaling pathways regulate the ALP core machinery through PTMs and transcriptional events. mTORC1, a serine/threonine kinase, is a master controller of the ALP that inhibits autophagosome formation (Kim et al., 2011; Park et al., 2016). On the other hand, the Unc-51 Like Autophagy Activating Kinase 1 (ULK1) complex, a serine/threonine protein kinase forms a multimeric protein complex, composed of mammalian ULK1, FIP200, Atg13 and Atg101, and has been described as a key kinase for autophagosome formation. Active mTORC1 phosphorylates and inactivates the ULK1 complex, explaining in part its inhibitory role in the ALP. Nutrient starvation inhibits mTORC1 and activates the ULK1 complex, relocating this kinase to the pre-autophagosomal membrane structure (PAS) to promote the first steps of autophagosome biogenesis (Itakura and Mizushima, 2010). In this regard, it has been shown that Beclin-1, a subunit of the class III phosphatidylinositol 3-kinase complex (PI3K-III), is phosphorylated on S14 by the ULK1 complex in response to nutrient starvation and mTORC1 inhibition (Russell et al., 2013). Phosphorylated Beclin-1 activates the pro-autophagic PI3K-III complex and enhances the production of a specific pool of phosphatidylinositol-3-phosphate (PI3P), a phospholipid that functions as a signal for PI3P effectors, which allows for the recruitment of downstream autophagy core machinery components (Lu et al., 2011; Dooley et al., 2014). On the other hand, other protein groups are the MAP1LC3s (LC3A, LC3B and LC3C) and the Gamma-aminobutyric acid receptor-associated proteins (GABARAPs; GABARAP, GABARAP-L1, GABARAP-L2), ubiquitin-like proteins that are covalently conjugated to phosphatidylethanolamine (PE) on autophagosomal membranes and promote the formation, elongation and maturation of autophagosomes (Kabeya et al., 2004; Nakatogawa et al., 2007).

One intriguing question in this field is: What is the major membrane source for autophagosome biogenesis? Cell compartments such as the Golgi apparatus (van der Vaart and Reggiori, 2010; Yamamoto et al., 2012), plasma membrane (Ravikumar et al., 2010), mitochondria (Hailey et al., 2010; Hamasaki et al., 2013) and endosomes (Puri et al., 2013) have been reported as membrane sources for autophagosome formation and elongation. Nevertheless, most of the evidence indicates that the endoplasmic reticulum (ER) is a key organelle that supplies a platform to the PAS. Upon nutrient starvation, PI3P is produced at specific sites on the ER that allows binding of the Double FYVE Containing Protein 1 (DFCP1), a protein implicated in the formation of the omegasome structure (Axe et al., 2008). Studies suggest that the omegasome acts as a platform for the recruitment of specific machineries implicated in the subsequent biogenesis of the isolation membrane or phagophore (Axe et al., 2008). Moreover, it has been shown that the mammalian small guanosine triphosphate hydrolase (GTPase) Rab1 and its yeast homolog Ypt1 regulate autophagosome formation (Zoppino et al., 2010). These GTPases are normally located in early secretory compartments such as the ER and Golgi apparatus, where their functions in protein trafficking is achieved (Plutner et al., 1991; Tisdale et al., 1992). In mammalian cells, an earlier report showed that GFP-Rab1B colocalizes with autophagosomes, which increases under nutrient starvation. Indeed, overexpression of GFP-Rab1B increased the number of LC3-positive autophagosomes (Zoppino et al., 2010). On the other hand, overexpression of the dominant negative mutant GFP-Rab1B N121I decreases LC3-positive autophagosomes (Zoppino et al., 2010). This data indicates that Rab1 could be considered as an important protein for the initial steps of autophagosome formation, mediating the early recruitment of the autophagy core machinery to the ER and Golgi apparatus. Supporting this data, a recent study found that Rab1B associates with Atg9A-positive vesicles, an autophagy related (Atg) transmembrane protein that controls the early steps of autophagosome biogenesis (Kakuta et al., 2017).

Until now few proteins have been reported as negative regulators of autophagy. Nevertheless, Rubicon (RUN domain-containing Beclin1-interacting protein) has been described as a suppressor of the later steps of autophagy. Early studies indicate that Rubicon is localized at endolysosomal structures where it binds to the PI3K-III complex, inhibiting its activity and impairing the maturation of autophagosomes and endolysosomal compartments (Zhong et al., 2009). Moreover, recent evidence showed that Rubicon regulates the levels of GTP-bound Rab7, a GTPase implicated in endosomal maturation and autophagy (Hegedűs et al., 2016), to avoid an exacerbated activity of Rab7 and to ensure endolysosomal homeostasis (Sun et al., 2010). These findings support the idea that Rubicon together with Rab7 must be considered as crucial regulators of autophagy, offering future therapeutic targets of intervention.

### ALP Regulation by Transcriptional Activity

Autophagosomes require fully active lysosomes to degrade their content. Moreover, in the last 8 years several studies revealed a key contribution of transcriptional activity in the ALP. Transcription factor EB (TFEB) and Transcription factor E3 (TFE3) are master regulators of lysosomal biogenesis and autophagy (Raben and Puertollano, 2016) and are members of the MiTF/TFE family of basic helix-loop-helix (bHLH) transcription factors. Furthermore, TFEB and TFE3 recognize a 10-base-pair motif (GTCACGTGAC) that is located at the promoter region of lysosomal and autophagy genes, a motif known as the coordinated lysosomal expression and regulation (CLEAR) element (Palmieri et al., 2011). Importantly, mTORC1 regulates the intracellular localization and transcriptional function of TFEB and TFE3. Under fully fed conditions active mTORC1 phosphorylates TFEB and TFE3 on S211 and S321, respectively, creating a binding site for 14-3-3 proteins that retain both transcription factors in the cytoplasm. Nutrient starvation triggers the inactivation of mTORC1 that subsequently promotes TFEB and TFE3 translocation to the nucleus to increase the expression of lysosome- and autophagy-related genes (Martina et al., 2012, 2014). Recently it was demonstrated that in response to nutrient starvation the calcium dependent serine/threonine protein phosphatase, calcineurin, is activated and dephosphorylates TFEB and promotes its nuclear translocation (Medina et al., 2015). These results indicate a concerted mechanism to induce expression of lysosomal genes that implicates mTORC1 inactivation and calcineurin activation. It also suggests that calcium release from intracellular compartments could lead to calcineurin-mediated ALP activation.

### Dysregulation of the ALP in AD and Its Molecular Players

Amyloid precursor protein (APP) is a type I transmembrane protein and is a substrate of two alternative proteolytic processing pathways. Amyloidogenic cleavage of APP by Beta-site APP cleaving enzyme 1 (β-secretase; also known as BACE1) generates CTFβ (also called C99; Burgos et al., 2010; Prabhu et al., 2012), that is subsequently cleaved by the γ-secretase complex producing Aβ (Vassar, 2004; Cole and Vassar, 2007). Non-amyloidogenic proteolytic processing occurs when APP is cleaved by A desintegrin and metalloproteinase domain-containing protein 10 (α-secretase; also known as ADAM10) producing the CTFα (also called C83) that is sequentially cleaved by the γ-secretase complex producing the non-toxic protein fragment p3 (Haass et al., 2012).

An early ultrastructural study showed that dystrophic neurites in human brains with AD are characterized by the accumulation of enlarged autophagosomes, amphisomes and endolysosomal organelles (Nixon et al., 2005). Similar observations were described in AD animal models, disturbances that occur at much earlier stages than Aβ deposition. Abnormal axonal endolysosomal structures contain decreased levels of luminal hydrolases (e.g., cathepsins B, D and L) strongly suggesting a decreased function of these organelles. Moreover, β-secretase levels are increased in these aberrant structures suggesting that production of Aβ could be enhanced at these sites (Gowrishankar et al., 2015).

Studies show that in neurons autophagosome biogenesis begins in distal axons and its maturation process occurs along the axon toward the cell body through a mechanism that depends on retrograde transport (Maday et al., 2012). Moreover, it has been proposed that autophagosomes fused with endosomes loaded on dynein motors in distal axons undergo retrograde transport towards the soma for content degradation (Cheng et al., 2015). Interestingly, in AD neurons amphisomes accumulate in axons probably due to a failure in this retrograde pathway. In this regard, intraneuronal Aβ oligomers disrupt dynein-driven retrograde transport of amphisomes, impairing degradation of their content in the soma of neurons (Tammineni et al., 2017). In addition, increased levels of APP and CTFβ also disrupt axonal retrograde trafficking and promote the appearance of enlarged endosomal structures in basal forebrain cholinergic neurons (Xu W. et al., 2016). Enhanced production of CTFβ develops axonal dystrophy in the absence of amyloid plaque (Rodrigues et al., 2012) and promotes an aberrant over-activation of the small GTPase Rab5, leading to pathologic characteristics such as endosome swelling (Kim et al., 2016). Because of the functional connection between Rab5 and Rab7 (Poteryaev et al., 2010), it is expected that Rab7 activity would also be increased by intracellular accumulation of CTFβ. Thus, emerging evidence reveals that CTFβ is a key toxic agent with deleterious effects in AD.

A growing body of evidence suggests that dysfunction of the autophagy-endolysosomal system precedes the accumulation of Aβ deposits (Cataldo et al., 2000; Wolfe et al., 2013). Indeed, Aβ is a normal component of brain physiology (Robakis, 2011) and neuroimaging studies have revealed Aβ deposits in cognitively normal individuals. Interestingly, some AD patients do not show Aβ deposits, which is evidence that debilitates the consensus that Aβ contributes to the early stages of AD (Edison et al., 2007; Li et al., 2008). Moreover, long-term potentiation (LTP) and cognitive impairments related to AD seem to correlate better with the intracellular accumulation of CTFβ, rather than with the presence of extracellular Aβ deposits (Jiang et al., 2010; Lauritzen et al., 2012).

APP and CTFβ together with β-secretase are delivered to endolysosomes through specific signal motifs to maintain proper protein levels and to avoid the deleterious effects of their accumulation (Burgos et al., 2010; Bustamante et al., 2013). In this regard, our group proposed that autophagosomes are key organelles for the degradation of CTFβ (and also CTFα) via the MVB-lysosomal pathway. We observed that inhibition of autophagosome biogenesis by depletion of Atg5 causes a strong accumulation of CTFβ in intra-luminal vesicles (ILVs) of MVBs. This was also observed when the fusion step between autophagosomes and the endolysosomal compartment was disrupted. In agreement with this evidence we showed that induction of autophagy by nutrient starvation and Torin1 treatment (an mTORC1 inhibitor) led to a reduction of CTFβ levels, confirming that autophagy contributes to the degradation of this fragment (González et al., 2017). Whether autophagy degrades CTFβ specifically or the entire endo/lysosomal organelle where CTFβ is enriched is not yet known. In agreement with these results neuronal-targeted TFEB overexpression in a mouse model of AD reduced APP and CTFβ steady-state levels, resulting in attenuated amyloid deposits. Moreover, in N2a neuronal cells TFEB-overexpression caused accelerated lysosomal degradation of APP/CTFs through endocytosis (Xiao et al., 2015). Furthermore, the involvement of the PI3K-III complex has been reported in the sorting of APP from the cell surface to endolysosomal and autophagic compartments (Swaminathan et al., 2016) and in the autophagic-lysosomal degradation of APP-CTFs in AD animal models (Wang et al., 2017; Yang et al., 2017). Altogether, APP-CTFs—but more importantly CTFβ—emerges as an important pathogenic factor for AD and as a potential biomarker in cerebrospinal fluid of AD patients (García-Ayllón et al., 2017).

Interestingly, the neuron specific DUB ubiquitin carboxyl-terminal hydrolase L1 (UCH-L1)—one of the most abundant proteins in the brain—is downregulated in sporadic AD brains (Choi et al., 2004) and its activity is reduced in the hippocampus of AD mice (Gong et al., 2006) providing strong evidence linking UCH-L1 to the pathogenesis of AD. Functionally, it has been proposed that UCH-L1 helps to maintain a stable pool of monoubiquitin during the ubiquitylation process (Tramutola et al., 2016). Indeed, dysfunction of UCH-L1 has been linked to several neurodegenerative diseases (Setsuie and Wada, 2007; Tramutola et al., 2016). Moreover, its hydrolase activity is essential for the maintenance of synaptic and cognitive function in mouse brains. Pharmacological inhibition of UCH-L1 impairs LTP (Gong et al., 2006), whereas its overexpression can rescue cognitive function in an AD mouse model (Gong et al., 2006; Zhang M. et al., 2014), however the mechanism is far from being elucidated. Because UCH-L1 activity has been demonstrated to be key in promoting the lysosomal degradation of β-secretase (Zhang et al., 2012) and thus, in the reduction of Aβ levels (Zhang M. et al., 2014), it would be interesting to explore the contribution of UCH-L1 activity in the ALP and its influence on CTFβ levels.

### Dysregulation of the ALP by the Neurotropic Herpes Simplex Virus Type 1 (HSV-1)

The herpes simplex virus type 1 (HSV-1) is a ubiquitous neurotropic virus that establishes lifelong latent infections in neurons and is a risk factor for neuronal damage and neurodysfunction in AD (Ando et al., 2008; Itzhaki et al., 2016; Otth et al., 2016; Itzhaki, 2017). Interestingly, new findings are beginning to reveal that the observed negative effects of HSV-1 neuronal infection could be linked with key players of the ALP (Ando et al., 2008; Conrady et al., 2010; Santana et al., 2012b; Harris and Harris, 2015).

The hypothesis connecting HSV-1 infection to AD gained importance due to the detection of viral DNA sequences in neurons (Saldanha et al., 1986; Leissring et al., 1998; Mori et al., 2004), as well as viral antigens in neurons and in intranuclear inclusion bodies of astrocytes from people who suffer from AD (Ludlow et al., 2016). Moreover, a high correlation between HSV-1 and higher levels of Aβ together with a misdistribution of APP in HSV-infected cells has been observed (Cheng et al., 2011; Bearer, 2012). In fact, studies show that HSV-1 neuronal infection regulates AMPK and mTORC1, two kinases with crucial roles in the ALP (Chuluunbaatar et al., 2010; Martin et al., 2014; Leyton et al., 2015; Vink et al., 2017). Nevertheless, how HSV-1 mechanistically perturbs the ALP is far from being elucidated. The first link between HSV-1 infection and the ALP was described in 1978 using electron microscopy (EM), observing HSV-1 virions inside autophagosomes (Smith and de Harven, 1978). Later it was found that ICP34.5, a HSV-1 protein that by itself causes neurovirulence in mice (Chou and Roizman, 1990), inhibits the anti-viral response (Tallóczy et al., 2002). Interestingly, ICP34.5 directly interacts with Beclin-1, a subunit of the PI3K-III kinase that is critical for autophagosomal formation (Chou and Roizman, 1990; Leib et al., 2009). The authors found that neuronal HSV-1 infected cells show a massive increase in autophagosomal-like structures, probably due to inefficient fusion of these organelles with lysosomes (Santana et al., 2012a; Katzenell and Leib, 2016). Considering that HSV-1 is a neurotropic virus that causes lifelong latent infection in host neurons as well as the abundant literature linking HSV-1 with AD pathogenesis, it would be interesting to explore whether HSV-1 could be linked to abnormal accumulation of autophagosomes in dystrophic neurites from post-mortem AD patients (Nixon et al., 2005; Nixon, 2007; González et al., 2017) and to define the precise molecular mechanisms involved.

### The UPS and ALP: Two Closely Related Pathways

The UPS and ALP pathways regulate proteostasis, constituting a single network to achieve protein balance (Balch et al., 2008). In fact, when the UPS is overwhelmed or is inhibited, the ALP instead eliminates aberrant proteins. Autophagosomes sequester these proteins and deliver them to lysosomes for degradation. The ALP by contrast to the UPS can degrade a much wider spectrum of substrates that tend to be bulkier, such as protein complexes, oligomers and aggregates, and even whole cellular organelles. Although the UPS and ALP were considered for a long time as independent mechanisms, a growing body of evidence indicates an intimate crosstalk and cooperation between both pathways (Korolchuk et al., 2010). As an example, these two pathways share common substrates, such as the case of the aggregate-prone protein α-synuclein, the major component of Lewy bodies in Parkinson Disease (PD), which is a substrate of the UPS and ALP (Bennett et al., 1999; Webb et al., 2003). Similarly, CTFβ and Aβ—two key harmful proteins for neuronal function—are eliminated by these two pathways (Bustamante et al., 2013; Xiao et al., 2015; González et al., 2017; Wang et al., 2017; Yang et al., 2017). Additionally, it has been reported that certain enzymes of the ubiquitylation machinery participate in both degradation pathways. The E3 ligase Parkin implicated in PD, conjugates K48 polyubiquitin chains in mitofusins MFN1 y MFN2, two proteasomal substrates (Chan et al., 2011), and modifies the mitochondrial protein VDAC1 with K27 ubiquitin chains to mediate mitochondrial degradation by the ALP (Geisler et al., 2010). In summary, disturbances in common molecular aspects of these pathways is particularly relevant to pathophysiological conditions that provoke the accumulation of aberrant proteins, such as in aging as well as in a variety of late-onset neurodegenerative disorders, including AD. Under these conditions, any decay in the UPS and ALP will affect normal cellular function and their ability to effectively counteract proteotoxic stressors (Vernace et al., 2007; Morimoto, 2008).

### Ubiquitin: A Key Unifying Signal in the Maintenance of Proteostasis

Ubiquitylation for many years has been studied as the universal signal for the elimination of substrates used for degradation systems. According to the “ubiquitin code” model (Komander and Rape, 2012) the type of ubiquitin-moiety on the substrate defines its specific route for degradation. While K48-linked polyubiquitin chains work as the canonical signal for proteasomal degradation, K63-linked polyubiquitin chains are thought to mediate degradation through the ALP (Grice and Nathan, 2016). In contrast to this hypothesis, recent findings propose that all chain types are capable of facilitating degradation by both pathways, indicating that the key determinant would be based on the monomeric or oligomeric physical states of these receptors rather than on a specific ubiquitin-moiety (Lu et al., 2017a,b). In this regard, several ubiquitin receptors that mediate the recognition of ubiquitylated cargoes in the UPS and ALP have been described (Cohen-Kaplan et al., 2016; Grice and Nathan, 2016; Yu and Matouschek, 2017). Proteasome receptors cannot oligomerize and bind with high affinity to polyubiquitin chains on soluble substrates rather than on aggregates (Lu et al., 2017a). By contrast, autophagy receptors gain higher affinity for polyubiquitin chains through their oligomerization, allowing for efficient removal of ubiquitylated aggregates and altogether ensuring the most suitable pathway for their degradation (Lu et al., 2017a,b). Interestingly, oligomerization of ubiquitin receptors is controlled by PTMs, which determine their efficient function (Pan et al., 2016). Among all PTMs, phosphorylation and ubiquitylation were recently characterized, showing great potential in proteostasis control.

### Role of the p62/SQTM1 Receptor in the UPS and ALP

p62/SQTM1, a multidomain protein, was the first autophagy receptor discovered in mammals and is essential for the clearance of ubiquitylated aggregates that accumulate upon UPS inhibition (Bjørkøy et al., 2005; Komatsu et al., 2007; Lin et al., 2013). Interestingly—and similar to what was found at the level of the proteasome—expression of p62/SQTM1 is upregulated by Nrf2 activation (Jain et al., 2010; Gao et al., 2014).

Recent studies described the key role of p62/SQTM1 upon combined knockdown of Rpn10 and Rpn13, two proteasome ubiquitin receptors (Demishtein et al., 2017). p62/SQTM1 interacts non-covalently with ubiquitin or polyubiquitin chains via its UBA domain (Vadlamudi et al., 1996; Seibenhener et al., 2004). Moreover, the Phox and Bem1 (PB1) domain found on p62/SQTM1 plays a crucial role in its oligomerization, a physical state that regulates its function as a ubiquitin receptor and the fate of the target substrates via autophagy or the UPS (Bjørkøy et al., 2005; Itakura and Mizushima, 2011). In fact, deletion of the PB1 domain prevents its self-association and reduces its ability to decrease protein aggregates (Bjørkøy et al., 2005). Interestingly, p62/SQTM1 through its PB1 domain interacts with the 19S RP of the proteasome, promoting substrate delivery to the proteasome (Seibenhener et al., 2004). On the other hand, the LC3-interacting region (LIR) of p62/SQTM1 mediates its association with proteins of the LC3 and GABARAP subfamilies on the autophagosome, a key step of substrate degradation (Bjørkøy et al., 2005; Komatsu et al., 2007; Pankiv et al., 2007; Cohen-Kaplan et al., 2016).

p62/SQTM1 function is highly controlled by PTMs. To date, eight different phosphorylation sites on p62/SQTM1 have been described on functional domains (Matsumoto et al., 2011). As an example, CK2 is known to mediate phosphorylation of p62/SQTM1 on S403 within the UBA domain, which increases its affinity for polyubiquitin chains and the entry of substrates into autophagosomes (Matsumoto et al., 2011). Interestingly, ULK1 also mediates phosphorylation of p62/SQTM1 on the residues S403 and S407, enhancing its affinity for polyubiquitin chains and clearance of aggregates by autophagy (Ro et al., 2014; Lim et al., 2015).

Moreover, p62/SQTM1 itself undergoes ubiquitylation on the residue K420 within its UBA domain upon proteasomal inhibition, a modification that positively modulates its function as an autophagy receptor (Lee et al., 2017; Peng et al., 2017). In contrast, ubiquitylation of the K7 residue within its PB1 domain abrogates its oligomerization and its ability to reduce protein aggregates (Pan et al., 2016). Interestingly, p62/SQTM1 can also form oligomers with other ubiquitin receptors such as neighbor of BRCA1 gene 1 (NBR1), a protein that contains similar interacting motifs to p62/SQTM1 (Kirkin et al., 2009; Waters et al., 2009). Altogether this opens another level of complexity to the process, where the regulation of the NBR1 function by PTMs has only recently started to be characterized (Nicot et al., 2014).

Another question in this field is whether the UPS could be affected by ALP dysfuntion. Interestingly, knock out (KO) mice deficient in the ALP (KO Atg7) accumulate not only aggregates but also soluble UPS substrates without obvious alterations in proteasomal activity (Komatsu et al., 2006; Korolchuk et al., 2009). Because autophagy-deficiency (KO Atg7, KO Atg5 and KO FIP200) is accompanied by higher levels of p62/SQTM1 in different cell types, it has been suggested that autophagy inhibition has a negative effect on the UPS (Komatsu et al., 2007; Mathew et al., 2009; Wei et al., 2011). Interestingly, p62/SQTM1 knockdown rescues the levels of soluble UPS substrates under ALP inhibition, suggesting that high levels of p62/SQTM1 could mediate, in part, the disruption of UPS function (Korolchuk et al., 2009). However, whether the interaction of p62/SQTM1 with the proteasome is perturbed in the absence of the ALP is yet unknown. Moreover, how different kinases or DUBs implicated in the control of proteasomal function are affected upon ALP deficiency is completely unexplored. Any deficiency in these activities could contribute to the increase of soluble UPS substrate levels in Atg7 KO mice. Finally, an increase in protein aggregate levels has been shown to block the UPS by physical interaction (Mayer et al., 1996; Almeida et al., 2006; Tseng et al., 2008; Park et al., 2009; Lee M. J. et al., 2013), which is an aspect that should be considered.

### Role of Ubiquilins in the UPS and ALP

Ubiquilins (UBQLNs) are proteins that function as ubiquitin receptors regulating the degradation of ubiquitylated proteins in eukaryotes. Humans have five ubiquilin genes, each encoding a separate protein (UBQLN1-4 and UBQLNL). They are characterized by having an N-terminal UBA domain, that in some UBQLNs are separated by a variable middle region containing internal STI1 repeats, which are domains involved in protein-protein interactions (Marín, 2014). The UBL domain and STI1 repeats in UBQLNs allow them to bind components of the UPS (Hjerpe et al., 2016) and ALP (N’Diaye et al., 2009; Rothenberg et al., 2010; Lee D. Y. et al., 2013), whereas the UBA domain participates in the binding of polyubiquitylated chains that are conjugated onto proteins marked for degradation. UBQLN1, UBQLN2 and UBQLN4 are expressed in all tissues, whereas UBQLN3 is exclusively expressed in testis (Conklin et al., 2000). Functional analysis demonstrated that mutations in UBQLN2 within its middle region leads to an impairment of protein degradation, linking this protein to defects in the proteostasis of brain tissue (Deng et al., 2011). In fact, it was recently demonstrated that UBQLN2 facilitates the clearance of protein aggregates through its interaction with the chaperones HSP70-HSP110 that facilitate protein disaggregation, shuttling ubiquitylated proteins for degradation through the UPS, a pathway that is functional even in ALP-deficient cells (Hjerpe et al., 2016). Moreover, UBQLN1, UBQLN2 and UBQLN4 participate in the ALP (N’Diaye et al., 2009; Rothenberg et al., 2010; Lee D. Y. et al., 2013). UBQLN4 interacts directly with LC3 through its middle region, mediating the recruitment of UBQLN1 to the autophagy machinery (Lee D. Y. et al., 2013). Altogether, this expands the roles of UBQLNs in protein degradation and opens interesting aspects that could be exploited. Interestingly, UBQLN1 has been shown to interact with APP through its middle STI1 repeats, preventing its accumulation and aggregation leading to the reduction of pathogenic species of Aβ (Stieren et al., 2011). Consistent with this, these authors demonstrated reduced levels of UBQLN1 in post mortem frontal cortex samples derived from late onset AD patients. Importantly, the interaction of UBQLN1 with APP is mediated by ubiquitylation of K688 within the cytosolic domain of APP (El Ayadi et al., 2012), demonstrating again that PTMs play key regulatory roles in protein homeostasis. In fact, our group demonstrated that mutation of all K residues within the cytosolic domain of APP leads to increased levels of CTFβ (Bustamante et al., 2013), an emerging pathogenic factor and a potential future biomarker for AD. Further studies will clarify whether UBQLNs could play unexplored roles in the clearance of APP and/or CTFβ through the UPS or ALP.

### The Role of Rpn11 in the Clearance of Aggresomes by the ALP

Aggresomes are perinuclear inclusion bodies located close to the microtubule-organizing center (MTOC; Kawaguchi et al., 2003). They play a protective role when cells face overloading of abnormal or damaged proteins that cannot be efficiently eliminated by the UPS. Despite the role of Rpn11 in the UPS where it catalyzes the deubiquitylation of the vast majority of the proteins that enter the proteolytic chamber of the 20S proteasome, normally coupled to K48-linked polyubiquitin chains, recent findings indicate that Rpn11 also participates in the clearance of protein aggregates via the ALP. Consistent with these findings, Rpn11 expression was shown to be highly reduced in human AD brain tissue, suggesting that it has a crucial function in neuronal proteostasis (Puthiyedth et al., 2016). Removal of ubiquitin chains by Rpn11 in aggresomes produces a large number of free unanchored K63-ubiquitin chains, molecules that surprisingly seem to be critical for coordinating the elimination of protein aggregates through the ALP (Hao et al., 2013; Nanduri et al., 2015; Mevissen and Komander, 2017). Free unanchored K63-ubiquitin chains activate the non-canonical function of HDAC6, currently known as a histone deacetylase. These chains trigger the interaction of HDAC6 with dynein motors, allowing for the efficient recruitment of protein aggregates and their transport along microtubules redirecting them to the MTOC to form aggresomes (Kawaguchi et al., 2003). Moreover, free unanchored K63-ubiquitin chains are key molecules for efficient autophagosome-lysosome fusion at the MTOC, a key step for final degradation of protein aggregates (Hao et al., 2013; Nanduri et al., 2015). HDAC6 deacetylates the cytoplasmic protein cortactin increasing its ability to interact with F-actin, which contributes to the assembly of the cortactin/F-actin network, which is necessary for autophagosome-lysosome fusion (Pandey et al., 2007; Lee J. Y. et al., 2010). In addition, the E3 ligase Tripartite motif-containing 50 (TRIM50) colocalizes with aggresomes decorated with HDAC6 and p62/SQTM1 under proteasomal inhibition. Moreover, HDAC6 interacts with TRIM50 catalyzing its deacetylation at the residue K372 to regulate the shuttle of TRIM50 to aggresomes (Fusco et al., 2014). In agreement with these findings, deficiency of TRIM50 abrogates the clearance of aggresomes (Fusco et al., 2012). Therefore, collective data provide evidence regarding the central role of Rpn11 and free unanchored K63-ubiquitin chains in a cascade of events that regulate aggresome degradation via the ALP. Further studies will help to define the consequence of Rpn11 inhibition in brain function and possible regulatory mechanisms of its activity.

### Crossregulation of UPS and ALP Activity by Kinases

We have discussed the relevance of phosphorylation as a central hallmark in the control of proteostasis. Here, we discuss the role of selected kinases that regulate both the UPS and ALP. In particular, CK2, a serine/threonine kinase that positively regulates proteasomal activity (Castaño et al., 1996; Mason et al., 1996) has also been shown to mediate phosphorylation of p62/SQTM1 on S403 within the UBA domain. This increases its affinity for polyubiquitin chains and their degradation via the ALP, substrates that would otherwise accumulate due to their poor degradation by the UPS (Matsumoto et al., 2011). Another interesting kinase is CaMKIIα, which together with enhanced proteasomal activity (Djakovic et al., 2009; Bingol et al., 2010; Jarome et al., 2016) promotes phosphorylation of Beclin-1 on S90 and its K63-linked ubiquitylation, together favoring activation of the ALP (Li et al., 2017). Moreover, CaMKIIα phosphorylates O-GlcNAc transferase (OGT) on S20, which mediates the O-GlcNAcylation of the ULK1 complex and its positive role in autophagosomal biogenesis (Ruan et al., 2017). PKA activation on the other hand, which is known to enhance proteasome activity (Asai et al., 2009; Lokireddy et al., 2015; Guo et al., 2017) has an inhibitory role in the ALP. PKA phosphorylates LC3 on S12, inhibiting its processing and decreasing the number of autophagosomes (Cherra et al., 2010). Moreover, activation of PKA induces phosphorylation of p62/SQTM1 on S24, reducing its interaction with LC3 and altering the degradation of ubiquitylated substrates (Christian et al., 2014). Finally, p38 MAPK, which is known to mediate a negative regulatory role in proteasome activity is also a negative player in the ALP through different mechanisms. First, p38 MAPK phosphorylates Atg5 on T75, inhibiting its function and abrogating insertion of LC3 into autophagosomal membranes (Keil et al., 2013). Second, p38 MAPK phosphorylates ULK1, a modification that reduces its kinase activity and interrupts its association with Atg13, an essential subunit of the ULK1 Complex (He et al., 2018).

### Therapeutic Strategies for the Management of AD Through the UPS and ALP

The main goal of numerous studies is to discover pharmacological agents that can enhance the function of the two major catabolic pathways in eukaryotic cells, pathways whose function declines gradually during aging and age-related neurodegenerative diseases. Therefore, finding compounds that increase proteasome activity is a challenging and ongoing goal in biomedicine. Up to now, few compounds are known to activate proteasome activity *in vivo*. Sulforaphane increases proteasome levels *in vivo* through induction of the transcription factor Nrf2 (Liu et al., 2014). In addition, IU1 enhances proteasomal degradation by inhibiting USP14, a proteasome associated DUB (Lee B. H. et al., 2010). Finally, Rolipram, enhances 26S proteasome activity *in vivo* by activating PKA (Myeku et al., 2016).

Together with the proteasome, another well-investigated approach is to enhance the activity of the ALP. Among all autophagy inducers tested, Rapamycin, an inhibitor of the mTOR signaling pathway and a potent activator of TFEB can reduce Aβ levels in the brain of transgenic AD mice and abrogate the associated cognitive deficit (Spilman et al., 2010). Another interesting compound is Curcumin, a polyphenol antioxidant that is highly enriched in yellow curry. Similar to Rapamycin, Curcumin inhibits the mTOR signaling pathway and activates TFEB, attenuating the cognitive impairments and the generation of Aβ in AD models (Wang et al., 2014; Zhang et al., 2016). Trehalose, another attractive natural compound that is not synthesized in mammals and is widely used in food, increases the removal of pathogenic aggregates through the enhancement of autophagy (Sarkar et al., 2007). Indeed, trehalose reduces Aβ levels in the hippocampus (Du et al., 2013) and ameliorates the learning ability and spatial memory in two different transgenic AD mouse models (Du et al., 2013; Portbury et al., 2017). Finally, hyperforin and its derivative Tetrahydrohyperforin (IDN5706), an active component of the plant *Hypericum perforatum* that is better known as St. John’s Wort, prevent cognitive deficits and Aβ accumulation in an AD mouse model (Inestrosa et al., 2011), an effect mediated in part by ALP activation (Cavieres et al., 2015). Emerging evidence of proteasome and ALP activators offers potent therapeutic approaches to prevent or delay symptoms of AD pathogenesis.

## Concluding Remarks

**Figure 1:**
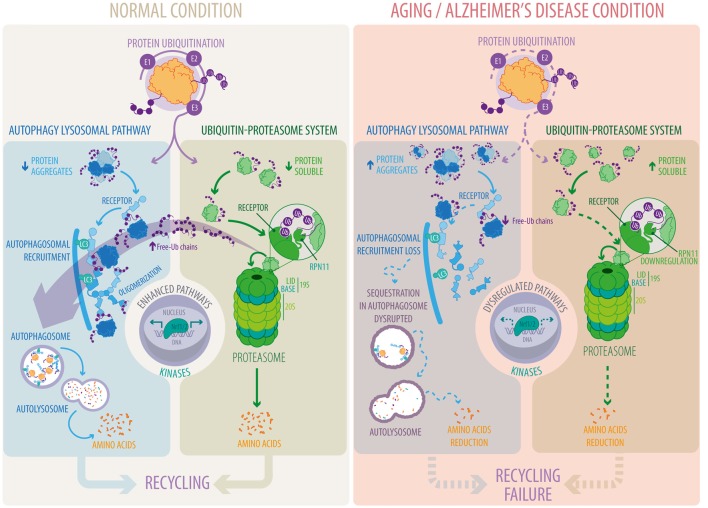
Autophagy-lysosomal pathway (ALP) and ubiquitin-proteasome system (UPS) pathways under normal and pathological conditions. Proteins are tagged with ubiquitin conjugates through a sequential enzymatic mechanism involving three classes of enzymes, E1, E2 and E3. *Under normal conditions*, ubiquitylated substrates are recognized by ubiquitin receptors present in ALP and UPS pathways and efficiently eliminated. In the UPS, substrates are subsequently deubiquitylated by RPN11, a key step for substrate degradation and amino acid recycling. Free-Ub chains formed by RPN11 activity promote ALP function. Ubiquitin receptors in the ALP, in contrast to the UPS, form oligomers to facilitate substrate recognition and autophagosomal recruitment. *Under aging and Alzheimer’s disease conditions* there is a decrease in the function of the ALP and the UPS that reduces substrate degradation and amino acid recycling. Downregulation of RPN11 in Alzheimer’s disease (AD) decreases free-Ub chains disrupting substrate recognition, their recruitment into autophagosomes and their final degradation by the ALP. Altogether, leading to the accumulation of deleterious protein aggregates. Transcriptional regulation (Nrf1/2) and phosphorylation (kinases/phosphatases) play a crucial role in ALP and UPS function whereas their dysregulation is the focus of intense studies in aging and Alzheimer’s disease.

The UPS and ALP are crucial pathways in proteostasis control, whose functions decline during aging and age-related diseases (Figure 1). A current fundamental challenge in biomedicine is the identification of novel approaches to promote activation of both systems to prevent accumulation of protein aggregates and their harmful deleterious effects. One interesting option is the phosphorylation of key proteins implicated in these pathways (Figure 1). Among all kinases involved, p38 MAPK and CaMKIIα stand out as attractive targets for drug discovery, mainly because of their positive impact on both pathways. Another interesting target is Rpn11, which releases free unanchored ubiquitin chains, a key step for protein entry into the proteolytic chamber of the proteasome (Figure 1). Interestingly, free unanchored ubiquitin chains are also crucial for the degradation of protein aggregates by the ALP. Therefore, enhancing the deubiquitinase activity of Rpn11 might a good strategy to increase the activity of the UPS and ALP. Recently it was reported that Rpn11 can be acetylated, ubiquitylated and phosphorylated^1^, however, the biological function of these PTMs has not been studied. It would be of great interest to evaluate whether any of these modifications increase Rpn11 activity. Finally, the Nrf1/2 transcription factors positively regulate proteostasis in response to metabolic and cellular stress conditions. Moreover, Nrf2 positively regulates p62/SQTM1 levels whereas p62/SQTM1 in turn activates the Nrf2 signaling pathway causing a positive feedback loop that leads to the clearance of protein aggregates. Therefore, activating the Nrf1/2 signaling pathway could be an attractive therapeutic target for the prevention of neurodegenerative diseases, including AD (Figure 1).

## Author Contributions

All authors contributed in the writing of this review.

## Conflict of Interest Statement

The authors declare that the research was conducted in the absence of any commercial or financial relationships that could be construed as a potential conflict of interest.

## Footnotes

^1^http://www.phosphosite.org

## Abbreviations

Atg: autophagy related
ATPase: Adenosine triphosphate hydrolase
GABARAP: Gamma-aminobutyric acid receptor-associated protein
GTPase: guanosine triphosphate hydrolase
KO: Knock out
LC3: Microtubule-associated protein light chain 3
MAPK1: Mitogen-activated protein kinase 1
Nrf1: Nuclear respiratory factor 1
Nrf2: Nuclear factor erythroid 2 like 2
PB1: Phox and Bem1 domain
Rab: monomeric GTPase/GTP-binding protein
Rpn: Regulatory particle of non-ATPase subunit
Rpt: Regulatory particle of triple-A subunit
TFE3: Transcription factor E3
TFEB: Transcription factor EB
TRIM50: Tripartite motif-containing 50
ULK1: Unc-51 Like Autophagy Activating Kinase 1.
